# Evaluating ‘enhancing pragmatic language skills for young children with social communication impairments’ (E-PLAYS): protocol for a feasibility randomised controlled trial study

**DOI:** 10.1186/s40814-019-0456-z

**Published:** 2019-06-08

**Authors:** Suzanne Murphy, Victoria Joffe, David Messer, Sarah Crafter, Jessica Radley, Sailaa Sunthararajah, Kerry Bell, Belen Corbacho, Caroline Fairhurst, Sara Rodgers, David Torgerson, Charlie Welch

**Affiliations:** 1 Institute of Health Research, University of Bedfordshire, University Square, Luton, LU1 3JU UK; 2 Division of Language and Communication Science, School of Health Sciences, University of London, Northampton Square, London, EC1V 0HB UK; 3 Education & Language Studies, Faculty of Wellbeing, Open University, Walton Hall, Kents Hill, Milton Keynes, MK7 6AA UK; 4 School of Psychology, Faculty of Arts & Social Sciences, Open University, Walton Hall, Kents Hill, Milton Keynes, MK7 6AA UK; 5 Department of Psychiatry, Warneford Hospital, Oxford, OX3 7JX UK; 6 Research and Development Office, North East London NHS Foundation Trust, Goodmayes Hospital, Barley Lane, Ilford, IG3 8XJ UK; 7 Department of Health Sciences, University of York, Heslington, YO10 5DD UK

**Keywords:** Social communication, Pragmatic language, Randomised controlled trial, Feasibility study, Young children, Peer collaboration, Communication impairment, Computer game

## Abstract

**Background:**

A number of children experience difficulties with social communication and this has long-term deleterious effects on their mental health, social development and education. The proposal presented in this article describes a feasibility study for a trial to test an intervention (‘E-PLAYS’) aimed at supporting children with social communication impairments. E-PLAYS harnesses technology in the form of a novel computer game in order to develop collaborative and communication skills. Preliminary studies by the authors show that when E-PLAYS was administered by the research team, children with social communication impairments showed improvements on communication test scores and on observed collaborative behaviours. The study described here is a pragmatic trial to test the application of E-PLAYS delivered by NHS speech and language therapists together with schools.

**Methods:**

This protocol outlines a two-arm feasibility cluster-randomised controlled trial of the E-PLAYS intervention with treatment as usual control arm, with randomisation at the level of the speech and language therapist. The aim of this study is to ascertain whether it will be feasible to progress to running a full-scale definitive trial to test the effectiveness of E-PLAYS in an NHS setting. Data relating to recruitment and retention, the appropriateness of outcomes and the acceptability of E-PLAYS to participants will be collected.

Speech and language therapists will select suitable children (ages 4–7 years old) from their caseloads and deliver either the E-PLAYS intervention (experimental group) or treatment as usual (control group). Assessments will include blinded language measures and observations, non-blinded teacher-reported measures of peer relations and classroom behaviour and parent-reported use of resources and quality of life. There will also be a qualitative process evaluation.

**Discussion:**

The findings of this study will inform the decision as to whether to progress to a full-scale definitive randomised controlled trial to test the effectiveness of E-PLAYS when delivered by speech and language therapists and teaching assistants within schools. The use of technology in game form is a novel approach in an area where there are currently few available interventions.

**Trial registration:**

ISRCTN 14818949 (retrospectively registered).

## Background

Children with social communication impairments experience difficulties with using linguistic context to understand speakers’ meanings and, more broadly, with applying conventional norms and expectations from the wider society to relate to others [1]. To illustrate, children with social communication impairments are likely to find it difficult to take turns during conversation, maintain a topic of conversation appropriately, recount coherent narratives, respond contingently, make inferences, understand non-literal language such as jokes, irony or sarcasm, repair communication breakdowns and generally follow conversational conventions. Pragmatic language skill may be defined as selection of the appropriate message or interpretation in relation to the communicative context [2]. The terms ‘social communication’ and ‘pragmatic language’ are commonly used to refer to similar skills.

Recent systematic reviews of interventions for ‘social communication impairments’ or ‘pragmatic language impairments’ [3, 4] have adopted broad definitions for these terms encompassing both verbal and nonverbal aspects of communication to include abilities such as facial expression recognition and production [5]. Whilst the terms ‘social communication’ and ‘pragmatic skill’ have often been used interchangeably in the research literature [1], for the current proposal, we follow the suggestion by Matthew et al. [6] and define ‘pragmatics’ more specifically as the *linguistic* component (excluding facial communication and other non-verbal communication) of social communication.

Children with social communication impairments are a heterogeneous group and encompass both clinical and non-clinical groups. Clinical groups include the categories of ‘Language Impairment’ (also commonly known as ‘Specific Language Impairment’ and, more recently, ‘Developmental Language Disorder’), ‘Social (Pragmatic) Communication Disorder’ and ‘Autism Spectrum Disorders’ as defined in the Diagnostic and Statistical Manual of Mental Disorders (DSM-V; [7]). ICD 10 [8] as well as listing a number of pervasive developmental disorders (autism, Asperger’s and other pervasive developmental disorders) also describes a number of developmental disorders of language. Children experiencing difficulties with social communication are not limited to those covered by these DSM-V and ICD 10 diagnoses, however. Social communication and pragmatic language difficulties have also been shown to be associated with other clinical populations, notably those with ADHD [9] or conduct disorder [10, 11] and non-clinical groups such as shy children [12, 13]. There are also indications that social communication disorders are severely under-diagnosed, particularly in children of low socio-economic status [10, 14].

Research concerning children identified as having pragmatic language difficulties demonstrates that effects are far-reaching. Recent studies have shown associations between pragmatic language difficulties and behavioural problems [15], especially of the disruptive, externalising kind. Healthcare costs (excluding education costs) have been shown to be 36% higher for 4-year-old children with language disorders than for typically developing children [16]. A number of studies have shown long-term negative effects: children with social communication impairments experience problematic peer relationships [17] and are commonly rejected and victimised by peers at school [18, 19]. Individuals diagnosed with pragmatic language impairments as children were found to experience continuing problems with establishing social relationships (such as friendships) later in adulthood [20].

A particularly important skill for successful adult functioning, to which social communication is central, is the ability to collaborate with others. Collaborative and team-building skills are recognised as vital to future adult employment and participation in society [21]. A number of collaborative activities between children (such as collaborative learning, cooperative learning or peer tutoring) now commonly take place in classrooms and have generally been judged to be beneficial in terms of improved learning (e.g., [22, 23, 24]). Collaborative working has also been shown to improve peer relations and facilitate children’s feelings of belonging [25, 26]. However, group and dyadic work is not invariably beneficial, only collaborations where children are actively engaged and that are characterised by high-quality questioning, explanation, clarification of ideas, discussion and generally positive affect have been found to be productive [21].

Given their difficulties, it is to be expected that children with social communication impairments will struggle with collaborative group working and recent studies do indeed bear this out. By comparison to typically developing children in collaborative contexts, they can be aggressive or withdrawn [27], show more irrelevant behaviours and share less [28], ignore others’ questions and requests and give poorer directions [29].

Interventions to support pragmatic language and collaborative working are limited. The use of technology and gaming has been highlighted as a positive tool for facilitating communication and collaboration with peers in children with communication difficulties (e.g. [30, 31]). The application of computer technology for collaborative games to support children’s communication has a number of unique advantages over non-technological approaches. Children with social communication impairments are frequently overwhelmed by the complexity and unpredictability of social interaction. Computer games designed for two or more children to play together can be formatted so that they provide a ‘scaffold’ that guides and structures the interaction between children (for a review, see [32]). Properly designed, use of a computer game can support communication by structuring collaboration between children so that they solve problems within a fun environment together in partnership [33–35]. Typically, in collaborative activities, children with social communication disorders either participate very little, lacking the confidence to become involved or, alternatively, may attempt to dominate and run the activity solely according to their own agenda [27]. Managing these behaviours presents a serious challenge for speech and language therapists, teachers and teaching assistants [36]. Technology can be used to regulate turn-taking, ensuring equal participation between all players and preventing exclusion of any children. Furthermore, rule enforcement and reward-giving (such as virtual ‘prizes’) by a computer, rather than an adult, is viewed by children as more consistent, fairer and less arbitrary [37]. The flexibility afforded can also be harnessed to sustain children’s interest and concentration by adding surprises, colourful animations and unusual sounds and keeping the game flowing at a suitable pace [31, 38].

We have developed and carried out initial testing for a computer game intervention which aims to improve pragmatic language skill and the ability to collaborate in children with social communication impairments. Preliminary efficacy testing for ‘E-PLAYS’ (formerly known as the ‘Maze Game’) is described in Murphy et al. [29, 39] on 32 children randomised either to the intervention or to usual school practice. Children who received the computer game intervention showed significant increases on communication test scores, in the use of high-quality questioning and listening skills and in their positive ratings about collaborative work with peers [39]. It is important to note that for these studies, the intervention was delivered to the children by a trained, post-graduate psychology research assistant who was supervised by the research team. This represents an ‘ideal’ scenario for intervention and it is likely that results would differ from implementation in ‘real-life’ by professionals who would not have the benefit of intensive supervision, time and training. Successful implementation by the National Health Service (NHS) speech and language teams throughout the UK would require the E-PLAYS intervention to translate to a setting in which speech and language therapists and teaching assistants could learn to use it together without research team input or supervision.

Following on from our preliminary work with E-PLAYS [29, 39] therefore, the aim of the present study is to establish the feasibility of running a full-scale clinical trial to evaluate its clinical- and cost-effectiveness when implemented within the National Health Service (NHS) in the UK. Speech and language therapists are employed by and work within the NHS, but their day to day work frequently takes place in the school setting, rather than in clinics, particularly for young children.

This feasibility study will explore potential challenges and aim to develop a protocol for a follow-on large-scale RCT. Specific objectives include testing the *recruitment procedure* and recording the number of participants recruited over time. An important aspect will focus on determining *treatment fidelity*; E-PLAYS will be delivered by teaching assistants who have been trained by NHS speech and language therapists. We will also aim to establish the *acceptability* of E-PLAYS with speech and language therapists and with teachers, teaching assistants, parents and children and also explore the acceptability of randomisation to a control group. We will also seek to determine the feasibility of collecting resource-use data and quality of life measures for the purpose of calculating relative *cost*-*effectiveness*.

## Method

### Design

The design is a two-arm feasibility cluster-randomised controlled trial (cRCT) with randomisation at the level of the speech and language therapist (SLT). Each participating child receiving the E-PLAYS intervention will be a child with social communication impairments. For each participating child, there will be an associated teacher and parent who will complete questionnaires, a speech and language therapist and teaching assistant who will deliver the intervention and a typically developing child from the same class who will partner the child to play the E-PLAYS game. For ease of reference, these will be called ‘participant groups’; each participant group comprises one child with communication impairment, plus one parent, one teacher, one SLT, one teaching assistant and one typically developing partner child. The randomisation will occur at the level of the SLT because once an SLT has become familiar with E-PLAYS, it is possible that their approach to children with social communication difficulties may be influenced. Therefore, SLTs will receive immediate training if randomised to the intervention group and if allocated to the control group, will receive training after all study measures have been completed.

Participating children in the intervention arm will receive the E-PLAYS intervention while participating children in the control arm will receive treatment as usual. The trial will be pragmatic in nature with the interventions being delivered in the way that they would be delivered within the NHS. The effectiveness of E-PLAYS will not be addressed in this feasibility trial but left to a subsequent full-scale RCT, with the key outcomes of the present study relating to the feasibility of conducting such an RCT. Assessment of these feasibility outcomes will inform whether a full-scale trial is viable and inform its design and implementation.

### Setting

We will recruit SLTs and suitable children on their caseloads from within North East London NHS Foundation Trust (NELFT) Speech and Language Services.

### Participants

Participants will comprise the following in a ‘participant group’ comprising one child with social communication impairment (SCI) with associated relevant others:

- SLTs from NELFT
- Participating children (ages 4–7 years old) with SCI as selected by SLTs ‘focal children’
- Teaching assistants trained to use E-PLAYS by participating SLTs
- Teachers who will complete pre-, post- and follow-up test measures relating to participating children
- ‘Partner’ children; classmates for the focal children for the E-PLAYS intervention who will play the dyadic collaborative computer games that comprise E-PLAYS with them.
- Parents of the focal children who will complete post-test measures for health economics.

### Selection, recruitment and consenting of participants

The procedure for distribution of information sheets and consent forms for the different kinds of participants is described below. In all cases, potential participants will be given at least 48 h in which to make a decision.

- SLTs from NELFT

The research team will approach suitably employed paediatric SLTs, that is SLTs working with children aged 4- to 7-years-old attending mainstream (not special) schools. The SLTs will be invited to a presentation by the team, will have the opportunity to ask questions about the study and will be given information sheets and consent forms to take away to read. Consent forms can be returned by post, electronically or by hand to the research team. SLTs not responding within a week will be reminded and invited again by email once more only.

(b) Participating children with social communication impairments (SCI) ‘focal children’.

SLTs will review their caseloads and identify suitable children using the Social Communication Behaviour Checklist devised by Adams et al. [40]. The checklist requires the SLT to decide on the applicability of each of the following five statements to a given child:

- The child has trouble understanding and interpreting the social context and friendship, e.g. social roles, emotions
- The child has trouble understanding and/or using non-verbal aspects of communication, e.g. facial expression, intonation
- The child has trouble with aspects of conversation, e.g. beginning and ending, taking turns, giving relevant and sufficient information
- The child makes bizarre, tangential or inappropriate comments
- The child has difficulty using and understanding non-literal language

Children who are judged as having at least two of the five statements applying to them will be considered eligible for inclusion in the study provided they also meet the following additional criteria:

- Four- to seven-years-old at the time of the SLT’s assessment of eligibility
- Have at least minimum levels of English (including any children with English as an additional language)
- Not suffering with a hearing, visual or physical impairment severely affecting speech production

Once eligibility is confirmed, the research team will provide schools with participant information sheets to send to parents, one written for parents and one written in simpler terms for the child to share with their parent, as well as a consent form for parents to sign.

(c) Teaching assistants trained to use E-PLAYS by participating SLTs

Once the children with SCI have been selected by SLTs and consent has been received from parents, the teaching assistants who are supporting these children will be invited to take part in the study and will receive written information sheets and consent forms at school from the research team.

(d) Teachers who will complete pre-, post- and follow-up test measures relating to participating children

Teachers of recruited children will also be invited to take part in the study and will receive written information sheets and consent forms at school from the research team.

(e) ‘Partner children’ for focal children for the E-PLAYS intervention

‘Partner children’ will be suggested by teachers and teaching assistants in consultation with the relevant SLT. These children will be typically developing children without language disorders in the same class as the focal children. E-PLAYS comprises a series of dyadic computer games, some of which are played with an adult and some with the partner child. The importance of the role that peers can play in interventions has been the subject of a recent systematic review by [41]). These authors concluded that the inclusion of peers was one of the most promising strategies for intervention and resulted in positive impacts on social communication. An important aspect of peer-mediated interventions concerns the peers chosen to interact with focal children. These peers can provide appropriate language models and also an opportunity for children with SCI to practice newly acquired skills. In general, it has been reported that teachers children who are popular, prosocial, and self-confident as peer partners for interventions [42]. Locke et al.’s longitudinal study [42] did not report any adverse outcomes for typically developing children as a result of participating in a peer-mediated intervention. An important feasibility question will concern recruitment of these children and willingness of parents to allow participation. Schools will send participant information sheets to parents of the peer partner children, one written for parents and one written in simpler terms for the child to share with their parent (as above for children with social communication impairment), as well as a consent form for parents to sign.

(f) Parents of the children with SCI who will complete post-test measures for health economics.

Parents will be asked to consent to completion of these forms at the same time as they will be asked to provide consent on behalf of their children (b) above.

Whilst research assistants will pass on information sheets to participants via schools and request the return of consent forms, all participants will be given the option to contact the Chief Investigator in the event of additional questions. All participants will be free to withdraw at any time from the protocol treatment without giving reasons and without affecting their usual standard of care. The Chief Investigator will preserve the confidentiality of participants taking part in the study and is registered under the Data Protection Act.

### Randomisation and allocation process

Participating children with SCI will be cluster-randomised at the level of the SLTs. SLTs will be randomised 1:1 to receive either immediate briefing from the research team together with the manual on the E-PLAYS intervention or at to receive this at the end of the trial. Randomisation will take place after SLTs have consented to participate in the study, and have identified and recruited children on their caseloads to participate in the trial but before they receive briefing. Allocation will be via minimisation to ensure balance across the two groups based on the borough of the SLT (there are five boroughs within the district covered by NELFT, each served by a different SLT team) and number of children recruited (dichotomised around the median number of children recruited to form a two level factor). All SLTs will be randomised together as opposed to having a rolling recruitment/randomisation period. The minimisation will be implemented by the trial statistician at the York Trials Unit using MinimPy version 0.3. Once the allocations have been generated, they will be communicated to members of the research team responsible for training the SLTs, while ensuring that the outcome assessors (research assistants) remain blind to these allocations.

### Blinding

Parent- and teacher-completed measures cannot be blinded; however, measures collected by research assistants (RAs) will be blinded. Teachers and teaching assistants at the schools will be reminded not to reveal allocations to the research assistants at every visit. For the qualitative data collection (Fun Tool Kit, open-ended training questionnaires, observations and focus groups) in which blinding is not possible, research assistants will not collect data from the same schools in which they are collecting quantitative data in order to preserve blinding.

### Training

The SLTs in the intervention group will receive training from the research team and will then, in turn, train teaching assistants in schools to deliver the E-PLAYS intervention to the children. Training for SLTs will be kept to the minimum as the aim of this feasibility study is to explore the feasibility of implementing E-PLAYS within the NHS nationally without research team support. Therefore, SLTs will be introduced to the E-PLAYS game and to the manual only. The SLTs will use a training manual with which to train teaching assistants which covers the delivery of E-PLAYS in depth. They will train teaching assistants for around 1 h.

### Quantitative outcome measures

In addition to basic demographic data on age, gender and ethnicity, selected quantitative measures have been chosen for sound psychometric properties (validity, reliability, internal consistency, inter-rater reliability). The specific feasibility outcomes to ascertain recruitment, measure treatment fidelity, test acceptability of both the intervention and of the measures used, and test the feasibility of data collection for language and communication measures, social and mental health measures, generalisation and cost effectiveness for an economic evaluation are given below, with details of the instruments, scales, assessments and interviews to be used:

#### (a) Recruitment measures

A recruitment log will be kept to determine participation, drop-out and completion rates to inform a possible future full-scale trial.

#### (b) Language and communication outcome measures

##### (i) The Children’s Communication Checklist-2, (CCC-2, [43])

The CCC-2 is the most widely used, standardised questionnaire of communication impairment in research and clinical contexts and will be completed by teachers.

##### (ii) Test of Pragmatic Skills, (TPS, [44]).

This is an observational elicitation measure. The tester (RA) engages the child in structured play in which test questions are embedded. The TPS was a sensitive indicator and successful at detecting improvements in communication in our pilot study; it has been standardised on 650+ children by the author. It will be administered by blinded RAs.

##### (iii) Clinical Evaluation of Language Fundamentals-5 (CELF-5, [45]).

We will use one of the subscales; recalling sentences. This sentence repetition assessment is a frequently used measure and is generally regarded as a measure of overall language ability drawing upon a wide range of language processing skills [46]. This will also be administered by blinded RAs.

#### (c) Social behaviour, friendship and mental health measure

##### The Strengths and Difficulties Questionnaire

The Strengths and Difficulties Questionnaire (SDQ) is regarded as the ‘gold standard’ measure and is widely used as a mental health indicator with subscales assessing behavioural, emotional and peer problems. This will be completed by teachers [47].

#### (d) Measure of generalisation to social contexts

##### Dyadic collaborative construction task (Magformers®)

A frequently reported issue with interventions targeting children with SCI is that the skills learned do not generalise to contexts beyond those of the intervention. In order to explore whether communication skills learned through E-PLAYS do in fact generalise to other contexts, children will be video-recorded undertaking a typical classroom collaborative task with a peer. The task we will ask the children to complete involves constructing a Magformers® model following a set of video-recorded instructions; this will take around 10 min. Magformers® are plastic, brightly coloured, magnetised blocks (similar in size to Lego® but different in shape and operation) with which it is possible to construct small models (see magformers.co.uk). All children in intervention and control groups will undertake this task and be recorded; children’s use of different kinds of communication will be observed and coded from the video-recording. The Magformers® construction task will be facilitated and video-recorded by blinded RAs who will also undertake coding of the transcripts from the video-recordings.

#### (e) Health-economic measures

##### (i) Bespoke Questionnaire

A bespoke questionnaire designed by the team’s health economist based on previous work in studies with children will measure the child’s health services-use (parent-completed, non-blinded).

##### (ii) EQ-5D-Y [48], PedsQL [49]

Health utility: both the parent proxy EQ-5D-Y [48] and the parent proxy PedsQL [49] will provide measures of health-related utility.

For the feasibility study, we will not undertake a full economic evaluation; we will assess intervention and trial costs only. We will estimate research and implementation costs of undertaking a full trial.

### Data collection points for outcome measures

**Fig. 1:**
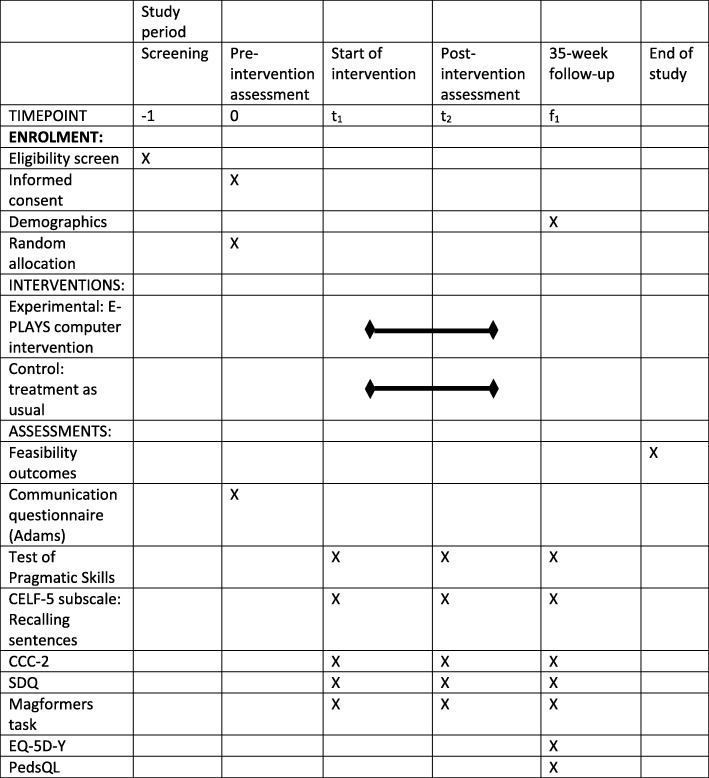
SPIRIT figure for E-PLAYS study

Data collection for the TPS, CCC-2, CELF-5 Recalling Sentences subscale, SDQ and Magformers® construction task will take place at baseline, 15–20 weeks and 35–40 weeks post-randomisation. These time points are equivalent to baseline, immediate post-intervention and 3-month follow-up for the intervention group. All health economic measures will be collected at 35–40 weeks post-randomisation (see Fig. 1. SPIRIT figure for E-PLAYS trial for data collection timings). Data will be collected at three times points from teachers, parents and the children themselves. As we are working with young children who may tire easily, measures will be collected in two to three short (< 30 min) sessions when necessary.

### Process evaluation

As recommended by the Medical Research Council (MRC) guidance for RCTs [50], we will supplement quantitative feasibility testing of E-PLAYS with a qualitative process evaluation which will examine the processes involved in intervention delivery. The processes we will explore are: whether instructions for delivery of E-PLAYS are adequate and clear; whether staff in a real-life NHS and school settings can use E-PLAYS faithfully; how acceptable staff find E-PLAYS; and whether children engage with and enjoy E-PLAYS. By conducting this process evaluation, we hope we may identify any possible influencing mechanisms or unintended negative effects not revealed by quantitative measures. In the event that, for example, we fail to recruit to target, or that fidelity appears compromised, the process evaluation may be illuminative. The process evaluation that forms part of this feasibility trial aims to investigate the following processes linked to implementation:

#### (a) Speech and language therapists’ and teaching assistants’ views

Focus groups with the speech and language therapists and teaching assistants who delivered E-PLAYS will be conducted once delivery is complete, and their views sought on acceptability and ease of use.

#### (b) Children’s views on E-PLAYS

The children’s view will be solicited using the Fun Toolkit [51] (which has been designed to measure children’s views of technology) by an RA immediately after each of two sessions.

#### (c) Fidelity evaluation

(i) On-line recording of the number of intervention sessions completed for each child and their content and duration in minutes will be automatically logged by the E-PLAYS software. For the E-PLAYS computer game, each child will have their own unique login, which will record the number of sessions undertaken, the time that they lasted and which parts of the programme were completed.
(ii) E-PLAYS sessions will be observed live by RAs to assess how faithfully teaching assistants actually use it in practice.
(iii) We will also explore fidelity issues in the focus groups described above.

#### (d) Training and instructions

**Fig. 2:**
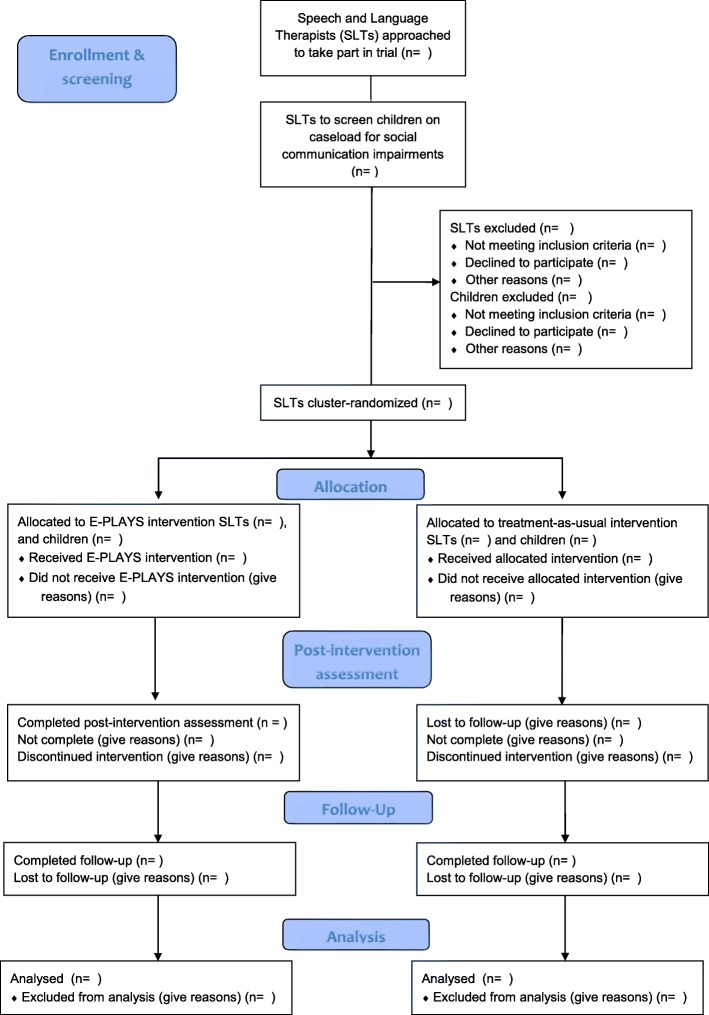
E-PLAYS study flowchart

Shortly after receiving the instruction pack and manual, SLTs will be asked to complete a short, open-ended questionnaire giving their impressions of its utility and clarity. Teaching assistants will receive a similar questionnaire after they have received the manual and instruction from SLTs (Fig. 2).

## Analysis

### Recruitment data analysis

The number of children recruited and the rate of attrition will be reported by allocation and in total. The number of E-PLAYS sessions completed and their duration will be summarised descriptively for children in the intervention arm.

### Analysis of outcome measures

A detailed analysis plan will be produced prior to the final analysis and will be reviewed by the independent trial steering committee. Analyses will follow intention to treat principles, with all data analysed as randomised. Analyses will be entirely descriptive with scores on the TPS, CCC-2, CELF-5 Recalling Sentences Subscale and Magformers® construction task presented unadjusted at each time point.

For the Magformers® construction task, video-recordings will be professionally transcribed and video-coding will comprise observation and categorisation of communicative (e.g. questions, directives, clarifications) and affective (e.g. positive and negative behaviours) items. The coding system is based on micro-analytic theory [52, 53] and was developed by the team specifically to analyse children’s collaborative interaction and has been shown in our studies to successfully detect changes in talk [29, 39, 54, 55]. The coding system incorporates elements from research into collaborative learning, conversation analysis and language impairment [29, 39, 53–60]. Video interaction will be coded by blinded RAs trained to fidelity by the Chief Investigator who has used this coding system extensively. RAs will train by watching and practice-coding training videos until they reach inter-rater reliability with these previously coded video-recordings. Areas of disagreement with the training videos will be discussed and explained by the Chief Investigator. Inter-rater reliability will further be tested between all RAs taking part in the study using a sample of 15% of all video-recordings.

The analysis of all measures will not be concerned with the effectiveness of the study intervention, but instead will seek to inform the feasibility objectives. In particular, this data will be used to characterise the groups at baseline, assess the proportion of analysable data at follow up and provide estimates of key population parameters, all of which will inform the design of a future full scale trial. The analysis of the health economic data will be principally concerned with the completeness and quality of this data. This will be reported together with a calculated estimate of the costs of running E-PLAYS within the NHS and schools and costs of a possible future full-scale RCT.

### Process evaluation analysis

#### (a) Speech and language therapists’ and teaching assistants’ views

The focus groups (a) will last around 1 h and will be audio-recorded and subsequently professionally transcribed. Nvivo 9 software will be used to aid a thematic analysis which will follow the guidelines of Braun and Clarke [61]:

(1) Becoming familiar with the data, (2) generating initial codes, (3) searching for themes, (4) reviewing themes and (5) defining and naming themes.

#### (b) Children’s views on E-PLAYS

Immediately after participating in sessions, children will be asked to rate their enjoyment and use of E-PLAYS using the Fun Toolkit [51]. The tool is easy to complete and requires no writing. It comprises a ‘Smileyometer’ scale; visual analogue scale with ‘smiley’ faces, ‘Fun Sorter’ (children sort which attributes of the game they like best) and ‘Again Again table’ (children rate which aspects of the game they would like to play again). The tool measures, besides user satisfaction, usability (in software terms) and ease of use. The Fun Toolkit has shown good test-retest reliability in previous research [51]. The Smileyometer, Fun Sorter and Again Again table produce numerical data for scoring; however, they are also designed to stimulate and encourage children to talk about the different aspects of the game and their enjoyment.

#### (c) Fidelity evaluation

##### (i) Number of intervention sessions

Descriptive statistics will be prepared of the number of intervention sessions completed for each child and their content and duration. The possible impact of treatment fidelity and adherence on outcomes will be explored.

##### (ii) Observations

Teaching assistants will be observed by RAs whilst delivering two sessions each to see how they actually deliver E-PLAYS in practice. A crucial aspect of the intervention is the extent to which the instructions in the manual are adhered to. Observers will also record instances of direct verbal feedback and children’s requests for help and comments.

The data from the open-ended questionnaire given to SLTs and teaching assistants will be analysed by coding responses into categories known as a ‘coding frame’ [62]. Given that this is a feasibility study and therefore exploratory in nature, the coding frame will be developed after the data is collected to cover a range of potential responses.

### Sample sizes

#### (a) Quantitative measures

It is not intended that this feasibility study will be powered to detect clinical differences between intervention and treatment as usual groups, this is left for the main trial. We are proposing to collect data on outcomes that will be used to inform a future large-scale trial. Sample sizes of between 24 and 70 have been recommended for feasibility trials to allow for the reliable estimation of a standard deviation for use in future sample size calculations [63, 64]. We therefore aim to recruit 70 children (35 in each group). We anticipate that attrition will be low; this is usually the case for school-based studies with young children, e.g. in our pilot study [39], we lost only one child over a follow-up period of 6 months. However, even with 20% loss to follow-up, we will retain 56 children, which is more than double the recommended minimum sample size. This number will also be sufficient to obtain a reasonably precise estimate of the intra-cluster correlation coefficient for the children grouped by SLT.

#### (b) Qualitative evaluation

All SLTs and teaching assistants in the intervention group (around seven speech and language therapists and 19 teaching assistants) will receive the open-ended questionnaire. Ten teaching assistants will be observed live delivering E-PLAYS and ten children will give their views using the Fun Toolkit. There will be four focus groups with four to five speech and language therapists or teaching assistants in each group.

## Discussion

The protocol outlined in this article describes a feasibility study for a trial to test an intervention (‘E-PLAYS’) aimed at supporting children with social communication impairments.

E-PLAYS uses novel computer technology to support and stimulate children to improve communication whilst collaborating with their peers. The protocol is based on our earlier pilot studies [29, 39], indicating that the intervention was efficacious when delivered by the research team. Whilst efficacy for E-PLAYS looks promising, it is important to remember that implementation in a ‘real-world’ context brings with it a number of challenges not immediately apparent in the closely controlled research setting of a pilot study. Thus, the study described here aims to investigate a number of important feasibility questions before proceeding to a full-scale trial. The context for the implementation of E-PLAYS is the NHS in the UK. NHS treatment for children with social communication disorders is the responsibility of SLTs. SLTs are specialist health professionals and frequently adopt a ‘consultation model’ whereby they provide training for teaching assistants in schools to deliver interventions rather than providing intervention directly themselves [65]. Therefore, E-PLAYS will fit well within typical NHS service delivery if it proves effective when provided via the consultation model and exploring the means of evaluating this is the aim of the present study.

The first aim of the feasibility study is to establish whether sufficient participants can be recruited for a full trial. Recruitment is complex, involving as it does a number of the adults concerned with each child. We will aim to determine whether sufficient numbers of SLTs, children and their parents, teachers and teaching assistants can be recruited and over what period of time for a definitive large-scale RCT.

The second major potential challenge to implementation in the NHS concerns fidelity. E-PLAYS is fully manualised, but is the manual sufficiently clear and comprehensive to ensure faithful delivery? Unlike in our previous pilot studies, should E-PLAYS be distributed nationally, it would not be viable for the research team to train numerous speech and language teams throughout the country. A key research task therefore involves investigating aspects of the indirect delivery whereby training will be provided to teaching assistants by SLTs. This differs fundamentally from delivery by a trained and supervised RA as in our pilot studies and feedback from SLTs and teaching assistants will be crucial.

Other challenges relate more broadly to difficulties with research concerning children with social communication impairments. SCI is notoriously difficult to measure [1] as it generally manifests itself only *during* dynamic social interaction, thus rendering testing with standardised questionnaires largely unachievable. A number of different approaches have been used to address this issue [1]. One approach is to ask teachers or another adult who knows the child to rate their pragmatic language. In this regard, the CCC-2 [43] is the most commonly used research measure. However, it is possible that global reports from adults may not be sensitive enough to detect changes resulting from interventions [40]. Furthermore, adult (particularly parent) report for conditions relating to autism and social communication may be especially susceptible to placebo effects [66, 67]. Another approach is to use an elicitation test, that is, to use a series of structured but naturalistic contexts which are designed to prompt particular communication behaviours for observation. The TPS is a well-validated elicitation measure for the age group targeted by E-PLAYS. Finally, observation and analysis of children’s spontaneous interactions using coding derived from conversation analysis [68] should provide an ecologically valid indication of any improvement. We will be video-recording children’s communications whilst playing with Magformers. The study will therefore incorporate all three of the approaches (adult report—CCC-2, TPS—elicitation test and video-observation) normally used to measure social communication to minimise the well-documented difficulties with measuring this construct. This is a particular strength of the study. In addition, the observational data from the Magformers task will provide a measure of generalisation, that is, an indication of whether the skills and strategies learned during the E-PLAYS intervention transfer to other, analogous collaborative contexts. Further, more distal measures of generalisation are included in the form of the SDQ which will measure the potential impact of communication changes on peer relations and classroom behaviour.

Ultimately, there is high potential for E-PLAYS to make a positive impact on children’s language learning. The intervention, being a computer game, can easily be scaled up and relies on simple technology available in all schools and NHS trusts. In the long-term, should the study progress to a full-scale RCT and demonstrate effectiveness, benefits to children with social communication impairments could be substantial and could include enhanced communication, more productive peer collaboration and improved classroom relations.

### Trial status

The current study status is that ethical approval was obtained on 4th December 2017 (Cambridge Central Research Ethics Committee, REC ref: 17/EE/0320, IRAS Project ID: 227864). The study opened to recruitment on 1st January 2018 and completed recruitment on 30th March 2018. Intervention delivery is currently proceeding.

## Abbreviations

CCC-2: Children’s Communication Checklist-2
CELF-5: Clinical Evaluation of Language Fundamentals-5
DSM-V: Diagnostic and Statistical Manual of Mental Disorders
E-PLAYS: Enhancing Pragmatic Language skills for Young children with Social communication impairments
EQ-5D: EuroQol 5-Dimension scale
MRC: Medical Research Council
NELFT: North East London NHS Foundation Trust
NHS: National Health Service
PedsQL: Paediatric Quality of Life inventory
SCI: Social communication impairment
SDQ: Strengths and Difficulties Questionnaire
SLT: Speech and language therapist
TA: Teaching assistant
TPS: Test of Pragmatic Skills

## References

[1] Norbury CF (2014). Practitioner review: social (pragmatic) communication disorder conceptualization, evidence and clinical implications. J Child Psychol Psychiatry.

[2] Bishop DVM (1997). Uncommon understanding: development and disorders of language comprehension in children.

[3] Parsons L, Cordier R, Munro N, Joosten A, Speyer R (2017). A systematic review of pragmatic language interventions for children with autism spectrum disorder. PLoS One.

[4] Wieckowski A, White S (2017). Application of technology to social communication impairment in childhood and adolescence. Neurosci Biobehav Rev.

[5] Insel T, Cuthbert B, Garvey M, Heinseen R, Pine DS, Quinn K (2010). Research domain criteria (RDoC): toward a new classification framework for research on mental disorders. Am J Psychiatr.

[6] Matthews D, Biney H, Abbot-Smith K (2018). Individual differences in children’s pragmatic ability: a review of associations with formal language, social cognition, and executive functions. Language Learning Dev.

[7] American Psychiatric Association (2013). Diagnostic and statistical manual of mental disorders.

[8] World Health Organisation. Multiaxial classification of child and adolescent psychiatric disorders. Cambridge: Cambridge University Press; 1996.

[9] Bishop DV, Baird G (2001). Parent and teacher report of pragmatic aspects of communication: use of the children’s communication checklist in a clinical setting. Dev Med Child Neurol.

[10] Donno R, Parker G, Gilmour J, Skuse D (2010). Social communication deficits in disruptive primary-school children. Br J Psychiatry.

[11] Gilmour J, Hill B, Place M, Skuse D (2004). Social communication deficits in conduct disorder: a clinical and community survey. J Child Psychol Psychiatry.

[12] Coplan RJ, Weeks M (2009). Shy and soft-spoken: shyness, pragmatic language, and socioemotional adjustment in early childhood. Infant Child Dev.

[13] Mewhort-Buist TA, Nilsen ES (2013). What are you really saying? Associations between shyness and verbal irony comprehension. Infant Child Dev.

[14] Bishop DVM, McDonald D (2009). Identifying language impairment in children: combining language test scores with parental report. Int J Lang Commun Disord.

[15] Ketelaars MP, Cuperus J, van Dall J, Jansonius K, Verhoeven L (2010). Pragmatic language impairment and associated behavioural problems. Int J Lang Commun Disord.

[16] Sciberras E, Westrupp EM (2015). Healthcare costs associated with language difficulties up to 9 years of age. Int J Speech Lang Pathol.

[17] Weismer SE, Cummings L (2013). Specific language impairment. Communication disorders.

[18] Laws G, Bates G, Feuerstein M, Mason-Apps E, White C (2012). Peer acceptance of children with language and communication impairments in a mainstream primary school: associations with type of language difficulty, problem behaviours and a change in placement organization. Child Lang Teach Ther.

[19] Mok PLH, Pickles A, Durkin K, Conti-Ramsden G (2014). Longitudinal trajectories of peer relations in children with specific language impairment. J Child Psychol Psychiatry.

[20] Whitehouse AJO, Watt HJ, Line EA, Bishop DVM (2009). Adult psychosocial outcomes of children with specific language impairment, pragmatic language impairment and autism. Int J Lang Commun Disord.

[21] Howe C (2010). Peer groups and children’s development.

[22] Azmitia M, Baltes PB, Staudinger UM (1998). Peer interactive minds: developmental, theoretical and methodological issues. Interactive minds: life-span perspectives on the social foundation of cognition.

[23] Christie D, Tolmie A, Thurston A, Howe C, Topping K (2009). Supporting group work in Scottish primary classrooms: improving the quality of collaborative dialogue. Camb J Educ.

[24] Johnson DW, Johnson RT (2009). An educational psychology success story: social interdependence theory and cooperative learning. Educ Res.

[25] Johnson-Pynn JS, Nisbet VS (2002). Preschoolers effectively tutor novice classmates in a block construction task. Child Study J.

[26] Tolmie A, Topping K, Christie D, Dolnaldson C, Howe C, Jessiman E (2010). Social effects of collaborative learning in primary schools. Learn Instr.

[27] Brinton B, Fujiki M, Montague EC, Hanton JL (2000). Children with language impairment in cooperative work groups: a pilot study. Lang Speech Hear Serv Sch.

[28] Kimhi Y, BaumingerZviely N (2012). Collaborative problem solving in young typical development and HFASD. J Autism Dev Disord.

[29] Murphy SM, Faulkner DM, Farley LR (2014). The behaviour of young children with social communication disorders during dyadic interaction with peers. J Abnorm Child Psychol.

[30] Holt S, Yuill N (2014). Facilitating other-awareness in low-functioning children with autism and typically-developing pre-schoolers using dual-control technology. J Autism Dev Disord.

[31] Ploog BO, Scharf A, Nelson D, Brooks PJ (2013). Use of computer-assisted technologies (CAT) to enhance social, communicative, and language development in children with autism spectrum disorders. J Autism Dev Disord.

[32] Fischer F, Kollar I, Mandl H, Haake JM (2007). Scripting computer-supported collaborative learning.

[33] Cress U, Wodzicki K, Bientzle M, Lingnau A (2011). Computer–supported collaborative learning for intellectually disabled pupils: stimulating interaction by using a floor control mechanism. Comput Support Collab Learn.

[34] Gal E, Lamash L, Bauminger-Zviely N, Zancanaro M, Weiss PL (2016). Using multitouch collaboration technology to enhance social interaction of children with high-functioning autism. Phys Occup Ther Pediatr.

[35] Piper AM, O'Brien E, Morris MR, Winograd TSIDES (2006). A cooperative tabletop computer game for social skills development. The 20th anniversary conference on computer supported cooperative work, 4–8 November Banff.

[36] Baines E, Blatchford P, Kutnick P (2009). Promoting effective group work in the primary classroom.

[37] Ben-Sasson A, Lamash L, Gal E (2013). To enforce or not to enforce? The use of collaborative interfaces to promote social skills in children with high functioning autism spectrum disorder. Autism.

[38] Grynszpan O, Weiss P, Perez-Diaz P, Gal E (2014). Innovative technology-based interventions for autism spectrum disorders: a meta-analysis. Autism.

[39] Murphy SM, Faulkner DM, Reynolds LR (2014). A randomised controlled trial of a computerised intervention for children with social communication difficulties to support peer collaboration. Res Dev Disabil.

[40] Adams C, Lockton E, Freed J, Gaile J, Earl G, McBean K (2012). The social communication intervention project: a randomized controlled trial of the effectiveness of speech and language therapy for school-age children who have pragmatic and social communication problems with or without autism spectrum disorder. Int J Lang Commun Disord.

[41] Chang Y-C, Locke J (2016). A systematic review of peer-mediated interventions for children with autism spectrum disorder. Res Autism Spectr Disord.

[42] Locke J, Rotheram-Fuller E, Kasari C (2012). Exploring the social impact of being a typical peer model for included children with autism spectrum disorder. J Autism Dev Disord.

[43] Bishop DVM (2003). The children’s communication checklist.

[44] Shulman BB (1986). Test of Pragmatic Skills–Revised.

[45] Wiig EH, Semel E, Secord WA (2013). Clinical Evaluation of Language Fundamentals®-Fifth Edition (CELF®-5).

[46] Klem M, Melby-Lerva M, Hagtvet B, Halaas Lyster S, Gustafsson J, Hulme C (2015). Sentence repetition is a measure of children’s language skills rather than working memory limitations. Dev Sci.

[47] Goodman R (2001). Psychometric properties of strengths and difficulties questionnaire. J Am Acad Child Adolesc Psychiatry.

[48] Wille N, Badia X, Bonsel G, Burström K, Cavrini G, Devlin N (2010). Development of the EQ-5D-Y: a child-friendly version of the EQ-5D. Qual Life Res.

[49] Varni JW, Seid M, Rode CA (1999). The PedsQL™: measurement model for the pediatric quality of life inventory. Med Care.

[50] Moore G, Audrey S, Barker M, Bond L, Bonell C, Hardeman W (2015). Process evaluation of complex interventions: Medical Research Council guidance. Br Med J.

[51] Read JC (2008). Validating the fun toolkit: an instrument for measuring children’s opinions of technology. Cogn Tech Work.

[52] Bull P (2002). Communication under the microscope: the theory and practice of microanalysis.

[53] Markell R, Asher S (1984). Children's interactions in dyads: interpersonal influence and sociometric status. Child Dev.

[54] Murphy S, Faulkner D (2006). Gender differences in verbal communication between popular and unpopular children during an interactive task. Soc Dev.

[55] Murphy S, Faulkner D (2011). The relationship between bullying roles and children's everyday dyadic interactions. Soc Dev.

[56] Anderson AH, Clark A, Mullin J (1994). Interactive communication between children: learning how to make language work in dialogue. J Child Lang.

[57] Gottman JM, Parker JG (1986). Conversations of friends.

[58] Kruger A (1993). Peer collaboration: conflict, co-operation or both. Soc Dev.

[59] Lloyd P, Boada H, Forns M (1992). New directions in referential communication research. Br J Dev Psychol.

[60] Radziszewska B, Rogoff B (1988). Influence of adult and peer collaborators on children’s planning skills. Dev Psychol.

[61] Braun V, Clarke V (2006). Using thematic analysis in psychology. Qual Res Psychol.

[62] Bryman A (2016). Social research methods.

[63] Julious SA (2005). Sample size of 12 per group rule of thumb for a pilot study. Pharm Stat.

[64] Teare DM, Dimairo M, Shephard N, Hayman A, Whitehead A, Walters SJ (2014). Sample size requirements to estimate key design parameters from external pilot randomised controlled trials: a simulation study. Trials.

[65] Ebbels Susan H., McCartney Elspeth, Slonims Vicky, Dockrell Julie E., Norbury Courtenay Frazier (2018). Evidence‐based pathways to intervention for children with language disorders. International Journal of Language & Communication Disorders.

[66] Lord C, Wagner A, Rogers S, Szatmari P, Aman M, Charman T (2005). Challenges in evaluating psychosocial interventions for autistic spectrum disorders. J Autism Dev Disord.

[67] Smith T, Scahill L, Dawson G, Guthrie D, Lord C, Odom S (2007). Designing research studies on psychological interventions in autism. J Autism Dev Disord.

[68] Sidnell J (2010). Conversation analysis.

